# Modified cell-permeable JNK inhibitors efficiently prevents islet apoptosis and improves the outcome of islet transplantation

**DOI:** 10.1038/s41598-018-29481-9

**Published:** 2018-07-23

**Authors:** Hirofumi Noguchi, Chika Miyagi-Shiohira, Yoshiki Nakashima, Nana Ebi, Eri Hamada, Yoshihito Tamaki, Kazuho Kuwae, Naoya Kobayashi, Issei Saitoh, Masami Watanabe

**Affiliations:** 1 Department of Regenerative Medicine, Graduate School of Medicine, University of the Ryukyus, Okinawa, 903-0215 Japan; 2 Okayama Saidaiji Hospital, Okayama, 704-8192 Japan; 3 Division of Pediatric Dentistry, Graduate School of Medical and Dental Science, Niigata University, Niigata, 951-8514 Japan; 4 Department of Urology, Okayama University Graduate School of Medicine, Dentistry and Pharmaceutical Sciences, Okayama, 700-8558 Japan

## Abstract

We previously reported that treatment with a JNK inhibitory peptide (11R-JNKI) prevents islet apoptosis and enhances the islet function *in vivo*. In the present study, we explored more efficient JNK inhibitors. The inhibition of the JNK activity by five types of deletion peptides in 11R-JNKI was investigated. One of the peptides, 8R-sJNKI(-9), significantly prevented JNK activation. At a concentration of 1 µM, 8R-sJNKI(-9) inhibited JNK activity similarly to 10 µM 11R-JNKI and the inhibition of the JNK activity by 10 µM 8R-sJNKI(-9) was significantly greater than that by 10 µM 11R-JNK. To evaluate the effects of 8R-sJNKI(-9), porcine islets were cultured with 1 µM of 8R-sJNKI(-9) or 8R-mutant sJNKI(-9) (8R-mJNKI(-9)). After 1 day of culture, the numbers of islets in the 8R-sJNKI(-9)-treated group was significantly higher than that in the 8R-mJNKI(-9)-treated group. After islet transplantation, the blood glucose levels reached the normoglycemic range in 58.3% of streptozotocin-induced diabetic mice in the 8R-sJNKI(-9) group and 0% of the mice in the 8R-mJNKI(-9)-treated group. These data suggest that 8R-sJNKI(-9) inhibits islet apoptosis and improves islet function.

## Introduction

Pancreatic islet transplantation efficiently restores euglycemia and corrects glycosylated hemoglobin in patients with type 1 diabetes^1–3^. However, successful transplantation is limited by donor shortage as well as by a high loss of islets during isolation^4^ and after transplantation^5^. Stress conditions generated throughout the islet isolation process initiate the activation of pro-inflammatory pathways and β cell destruction^6–9^. It has been shown that the signal transduction pathway of c-Jun NH_2_-terminal kinase (JNK) is preferentially activated in response to processing of islet isolation and to inflammation during islet transplantation^10–14^. Activation of JNK has been associated with the decreased expression of the insulin gene and insulin resistance^15,16^. Isolated islets have been exposed to several forms of stress such as pancreas preservation, islet isolation, and inflammation and glucose toxicity after transplantation.

We previously reported that treatment with a peptide inhibitor of JNK (11R-JNKI) during pancreas preservation before islet isolation, islet culture, or islet transplantation, prevents islet apoptosis and enhances the islet graft function *in vivo*^12,13,17^. In the present study, we explored more efficient and low-priced JNK inhibitors. We attempted the deletion of three arginine and two glycine-linker of N-terminal, and several amino acids of the C-terminal in 11R-JNKI.

## Results

### Transduction of the JNK inhibitory peptide into pancreatic β-cells

**Figure 1:**
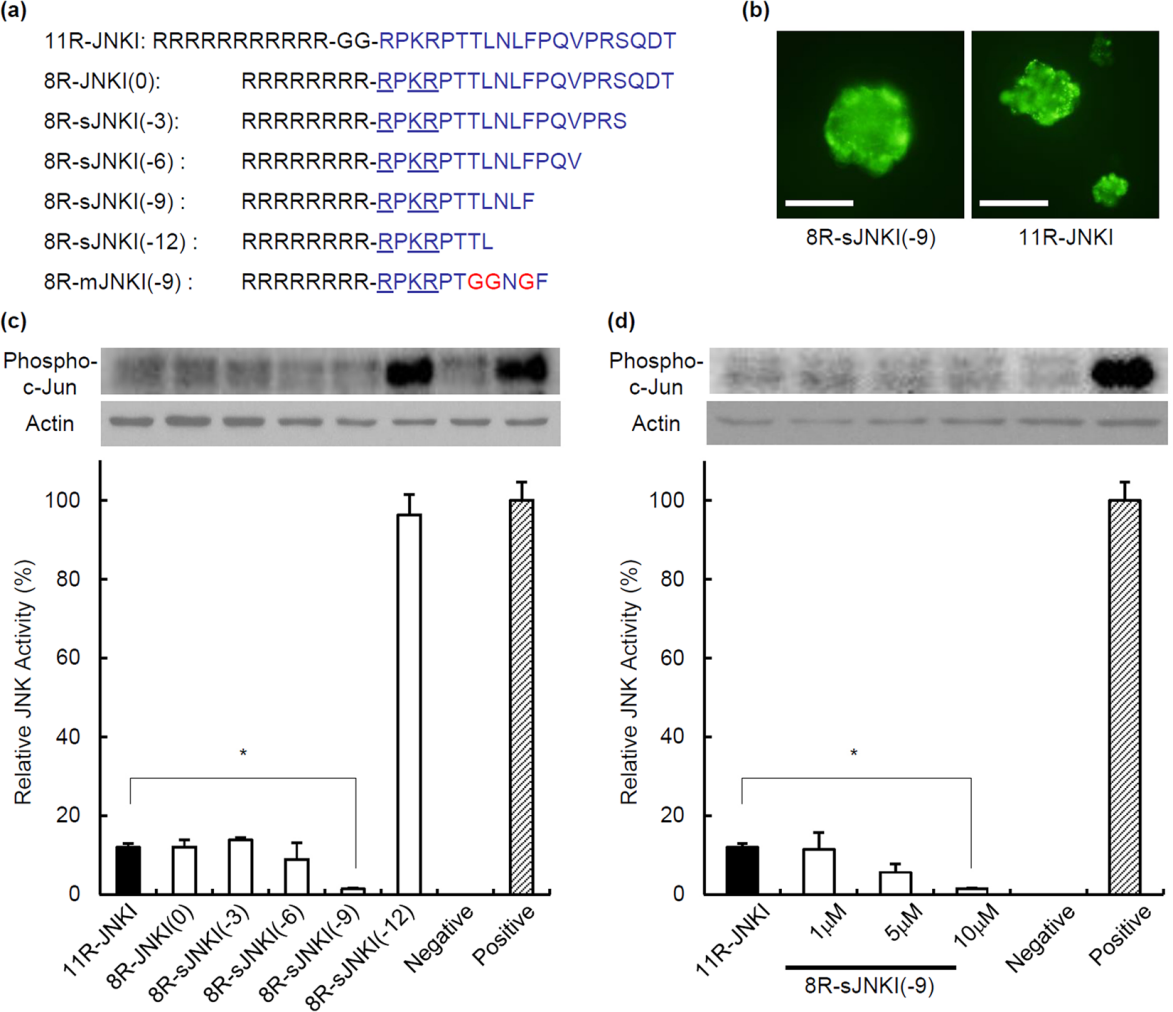
Transduction of 8R-sJNKI into pancreatic β-cells and its effect. (**a**) The sequences of the different peptides that were used. **(b)** Isolated islets were treated with 10 µM FITC-conjugated 11R-JNKI or 8R-sJNKI(-9). At 1 h after treatment, 11R-JNKI and 8R-sJNKI(-9) were observed as fluorescence signals in almost all of the islets. **(c)** Inhibition of stress-activated protein kinases, JNK activation. MIN6 cells were cultured with 10 µM of 11R-JNKI, 8R-JNKI(0), 8R-sJNKI(-3), 8R-sJNKI(-6), 8R-sJNKI(-9), or 8R-sJNKI(−12) for 23 h. The cells were then treated with 1 µg/mL anisomycin for 1 h to stimulate the activation of JNK, after which the JNK activity was examined. **(d)** The dose-response of 8R-sJNKI(-9). MIN6 cells were treated with 1–10 µM of 8R-sJNKI(-9) for 23 h. The cells were cultured with 1 µg/mL anisomycin to stimulate the activation of JNK for 1 h, after which the JNK activity was examined. Western blotting was performed using the rabbit anti-Phospho-c-Jun(Ser73)-specific antibody and mouse anti-β-actin antibody (control). The cell lysates from MIN6 cells cultured with and without 1 µg/ml anisomycin for 1 h were used as positive and negative controls, respectively. The data are expressed with the JNK activity of the positive and negative controls, which were arbitrarily set at 100 and 0, respectively. An asterisk indicates a significant difference between the two groups (p < 0.01).

We previously reported that treatment with a JNK inhibitory peptide (JNKI; RPKRPTTLNLF PQVPRSQDT) as a C-terminal fusion protein with 11-arginine (11R), which facilitates the uptake of peptides and protein into mammalian cells, inhibited islet apoptosis, and enhanced the islet function *in vivo*^12^. The N-terminal amino acids of JNKI include two arginine and one lysin (RPKR). Since poly-arginine/lysin facilitates the uptake of peptides and protein into mammalian cells^12,18–20^, we hypothesized that the transduction efficacy of 11R-JNKI may not be reduced after the deletion of three arginine and two glycine-linker. Moreover, we investigated whether C-terminal deletion peptides of JNKI can inhibit JNK activity (Fig. 1a). Isolated islets were treated with FITC-conjugated 11R-JNKI or 8R-smaller JNKI (sJNKI). At 1 h after treatment, 11R-JNKI and 8R-sJNKI were observed as a fluorescent signal in almost all of the islets (Fig. 1b). This finding shows that these peptides were efficiently delivered into the isolated islets.

To test whether 8R-sJNKI can prevent the activity of JNK, MIN6 cells (β-cell line) were cultured with 10 µM 8R-sJNKI for 23 h. MIN6 cells were then treated with anisomycin to stimulate the activation of JNK for 1 h. 8R-JNKI(0), 8R-sJNKI(-3), 8R-sJNKI(-6), 8R-sJNKI(-9),but not 8R-sJNKI(-12), significantly prevented JNK activation (Fig. 1c, Supplemental Fig. 1a). In particular, 8R-sJNKI(-9) peptide inhibited the JNK activity to 1–2% of the activity of untreated cells (Fig. 1c, Supplemental Fig. 1a). These findings show that 8R-sJNKI prevents JNK activation in cells.

To test the dose-response of 8R-sJNKI(-9), we treated MIN6 cells with 1–10 µM 8R-sJNKI(-9) for 23 h, followed by anisomycin for 1 h. 1 µM 8R-sJNKI(-9) inhibited the JNK activity similarly to 10 µM 11R-JNKI and the inhibition of the JNK activity by 10 µM 8R-sJNKI(-9) was significantly greater than the inhibition by 10 µM 11R-JNK (Fig. 1d, Supplemental Fig. 1b). The JNK activity of cells treated with 8R-sJNKI(-9) was inhibited in a dose-dependent manner (Fig. 1d, Supplemental Fig. 1b).

### Specificity of 8R-sJNKI(-9)

**Figure 2:**
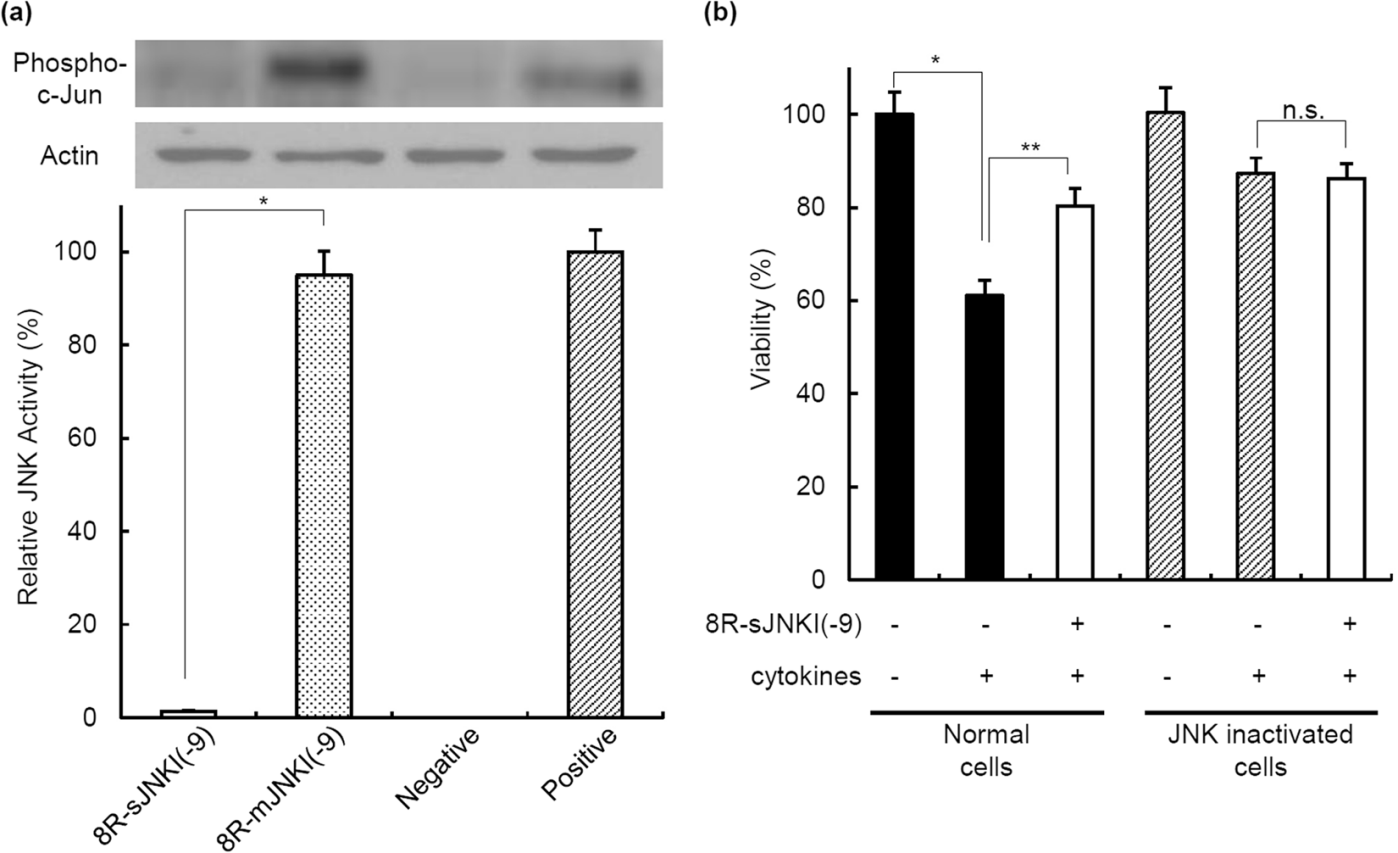
The specificity of 8R-sJNKI(-9). (**a**) The specificity of 8R-sJNKI(-9). MIN6 cells were cultured with 1 µM of 8R-sJNKI(-9) or 8R-mJNKI(-9) for 23 h. The cells were then treated with 1 µg/mL anisomycin to stimulate JNK activation for 1 h, after which the JNK activity was examined. Western blotting was performed using the rabbit anti-Phospho-c-Jun(Ser73)-specific antibody and mouse anti-β-actin antibody (control). The cell lysates from MIN6 cells treated with and without 1 µg/ml anisomycin for 1 h were used as positive and negative controls, respectively. The data are expressed with the JNK activity of the positive and negative controls, which were arbitrarily set at 100 and 0, respectively. An asterisk indicates a significant difference between the two groups (*p < 0.01). **(b)** The viability of normal and JNK-inactivated MIN6 cells after exposure of cytokines. MIN6 cells and JNK-inactivated MIN6 cells were cultured with 1 µM 8R-sJNKI(-9) for 23 h followed by exposure to a cocktail of pro-inflammatory cytokines (IL-1β, TNF-α, IFN-γ) for 18 h. Asterisks indicate a significant difference between the two groups (*p < 0.01, ** < 0.05).

To evaluate the specificity of 8R-sJNKI(-9), we synthesized 8R-mJNKI(-9), which has mutated sequences of JNKI(-9) peptide (Fig. 1a). MIN6 cells were cultured with 10 µM 8R-sJNKI(-9) or 8R-mJNKI(-9) for 23 h, followed by anisomycin for 1 h. 8R-sJNKI(-9) (1 µM), but not 8R-mJNKI(-9), inhibited the JNK activity (Fig. 2a, Supplemental Fig. 1c). We also used MIN6 cells in which JNK was inactivated by a genetic strategy (JNK1 siRNA). MIN6 cells and JNK-inactivated MIN6 cells were treated with 1 µM 8R-sJNKI(-9) for 23 h, followed by a cocktail of pro-inflammatory cytokines (IL-1β, TNF-α, IFN-γ) for 18 h. A 61.0% ± 3.2% reduction in MIN6 cell viability was observed after exposure of the cytokines, while MIN6 cells with 8R-sJNKI treatment were protected from this reduction. JNK-inactivated MIN6 cells appeared to be much more well protected against exposure to the cytokines than normal MIN6 cells. The viability of JNK-inactivated MIN6 cells with treatment of 1 µM 8R-sJNKI(-9) was similar to that without treatment of 1 µM 8R-sJNKI(-9) (Fig. 2b). These data demonstrated the specificity of 8R-sJNKI(-9).

### Effect of 8R-sJNK(-9) on isolated islets

**Figure 3:**
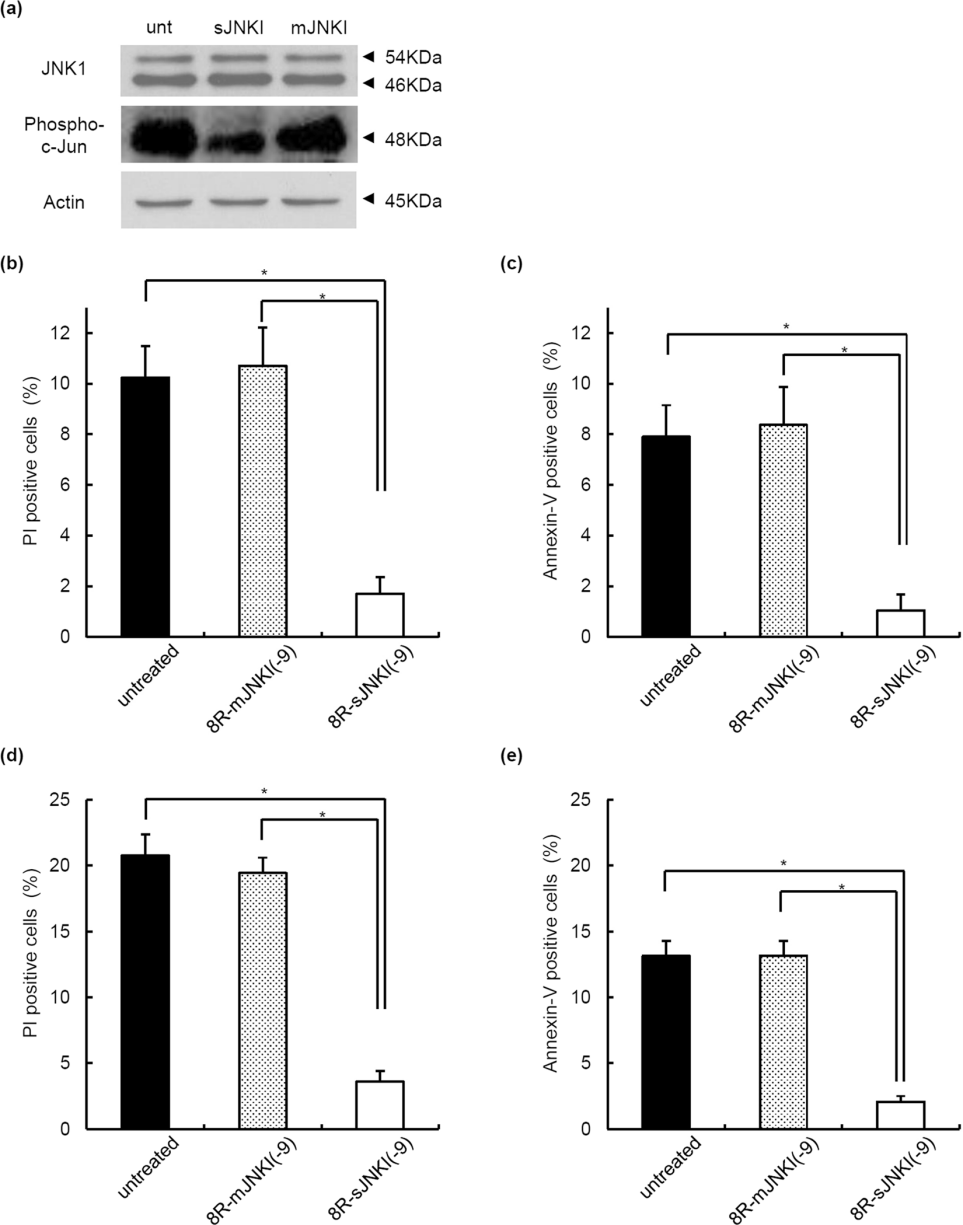
The effect of 8R-sJNKI(-9) on porcine islets. (**a**) Western blot. Cell extracts were fractionated by 10% SDS-PAGE and transferred to reinforced cellulose nitrate membrane. After blocking, the membranes were incubated overnight at 4 °C in TBS buffer containing a 1:1,000 dilution of rabbit anti-phospho-c-Jun antibody, mouse anti-JNK1 antibody, or mouse anti-β-actin antibody (control), and then incubated for 1 h at room temperature in TBS containing a secondary antibody. unt: untreated islets, sJNKI: islets treated with 8R-sJNKI(-9), mJNKI: islets treated with 8R-mJNKI(-9). **(b**,**c)** Propidium iodide (PI)/annexin-V assays. After 6-h culture with or without 1 µM of 8R-sJNKI(-9) or 1 µM of 8R-mJNKI(-9), 1,000 IE of islets were dispersed into single cells using Accutase and the single cells were then incubated with PI (**b**) and FITC-annexin-V (**c**). **(d**,**e)** Propidium iodide (PI)/annexin-V assays after exposure to cytokines. Islets that had been cultured for 48 h were treated with or without 8R-sJNKI(-9) or 8R-mJNKI(-9) for 23 h and then exposed to a cocktail of cytokines (IL-1β 50 U/mL, TNF-α 1,000 U/mL, IFN-γ 1,000 U/mL) for 18 h., 1,000 IE of islets were dispersed into single cells using Accutase, and the single cells were then incubated with PI (**d**) and FITC-annexin-V (**e**). The data are representative of six independent experiments.

We previously reported that JNK activity was relatively high after organ preservation and that it became progressively higher during islet isolation procedure^17^. To evaluate the effect of 8R-sJNKI(-9) on pancreatic islets, porcine islet isolation was performed (Tables 1, 2), and the isolated islets were cultured with or without 1 µM 8R-sJNKI(-9) or 8R-mJNKI(-9) for 6 h. 8R-sJNKI(-9) significantly inhibited the JNK activity, whereas 8R-mJNKI(-9) did not (Fig. 3a, Supplemental Fig. 1d,e). To examine whether or not treatment with 8R-sJNKI(-9) prevented islet apoptosis through JNK activation, propidium iodide (PI) and Annexin V assays were performed. Approximately 1,000 IE of islets after being cultured with or without 1 µM 8R-sJNKI(-9) or 8R-mJNKI(-9) for 6 h were dispersed using Accutase and the dispersed cells were then cultured with Annexin V and PI. The percentages of PI-positive cells in the no peptide, 8R-mJNKI(-9), and 8R-sJNKI(-9) groups were 10.2% ± 1.2%, 10.7% ± 1.5%, and 1.7% ± 0.6%, respectively (Fig. 3c). The percentages of Annexin V-positive cells in the no peptide, 8R-mJNKI(-9), and 8R-sJNKI(-9) groups were 7.9% ± 0.8%, 8.3% ± 0.8%, and 1.0% ± 0.4%, respectively (Fig. 3d). The apoptotic rate in the 8R-sJNKI(-9) group was significantly lower than that in the other two groups. These data suggest that treatment with 8R-sJNKI(-9) prevented apoptosis immediately after islet isolation.

**Table 1:** The characteristics of the tissue and procedures.

	n = 6
Pancreas size (g)	127.8 ± 7.5
Operation time (min)	6.0 ± 0.6
Warm ischemic time (min)	27.0 ± 0.9
Cold ischemic time (min)	1138.7 ± 9.7
Phase I period (min)	11.7 ± 0.6
Phase II period (min)	37.5 ± 1.1
Undigested tissue (g)	10.0 ± 1.4

The data are expressed as the means ± SE.

**Table 2:** The islet characteristics.

	n = 6
IE before purification	567,015 ± 135,277
IE after purification	433,544 ± 99,757
Embedded islets (%)	20.7 ± 3.3
Viability (%)	95.7 ± 0.4
Purity (%)	56.9 ± 7.0
Post-purification recovery (%)*	78.3 ± 6.8
Score	9.3 ± 0.2

The data are expressed as the means ± SE.

*Post-purification recovery (%) = IE after purification/(IE before purification) × 100.

To examine whether or not 8R-sJNKI(-9) prevented islet apoptosis induced by exposure to cytokines, islets that had been cultured for 48 h were treated with or without 1 µM 8R-sJNKI(-9) or 8R-mJNKI(-9) for 23 h and then exposed to a cocktail of pro-inflammatory cytokines (IL-1β, TNF-α, IFN-γ) for 18 h. 1,000 IE of islets were dispersed into single cells using Accutase, and the dispersed cells were then cultured with Annexin V and PI. The percentages of PI-positive cells in the no peptide, 8R-mJNKI(-9), and 8R-sJNKI(-9) groups were 20.8% ± 1.6%, 19.4% ± 1.2%, and 3.6% ± 0.8%, respectively (Fig. 3e). The percentages of Annexin V-positive cells in the no peptide, 8R-mJNKI(-9), and 8R-sJNKI(-9) groups were 13.1% ± 1.2%, 13.2% ± 1.1%, and 2.1% ± 0.4%, respectively (Fig. 3f). The apoptotic rate in the 8R-sJNKI(-9) group was significantly lower than that in the other two groups. These findings suggest that treatment with 8R-sJNKI(-9) prevented islet apoptosis after exposure to cytokines.

### The islet equivalents (IE), function, and ATP content

**Figure 4:**
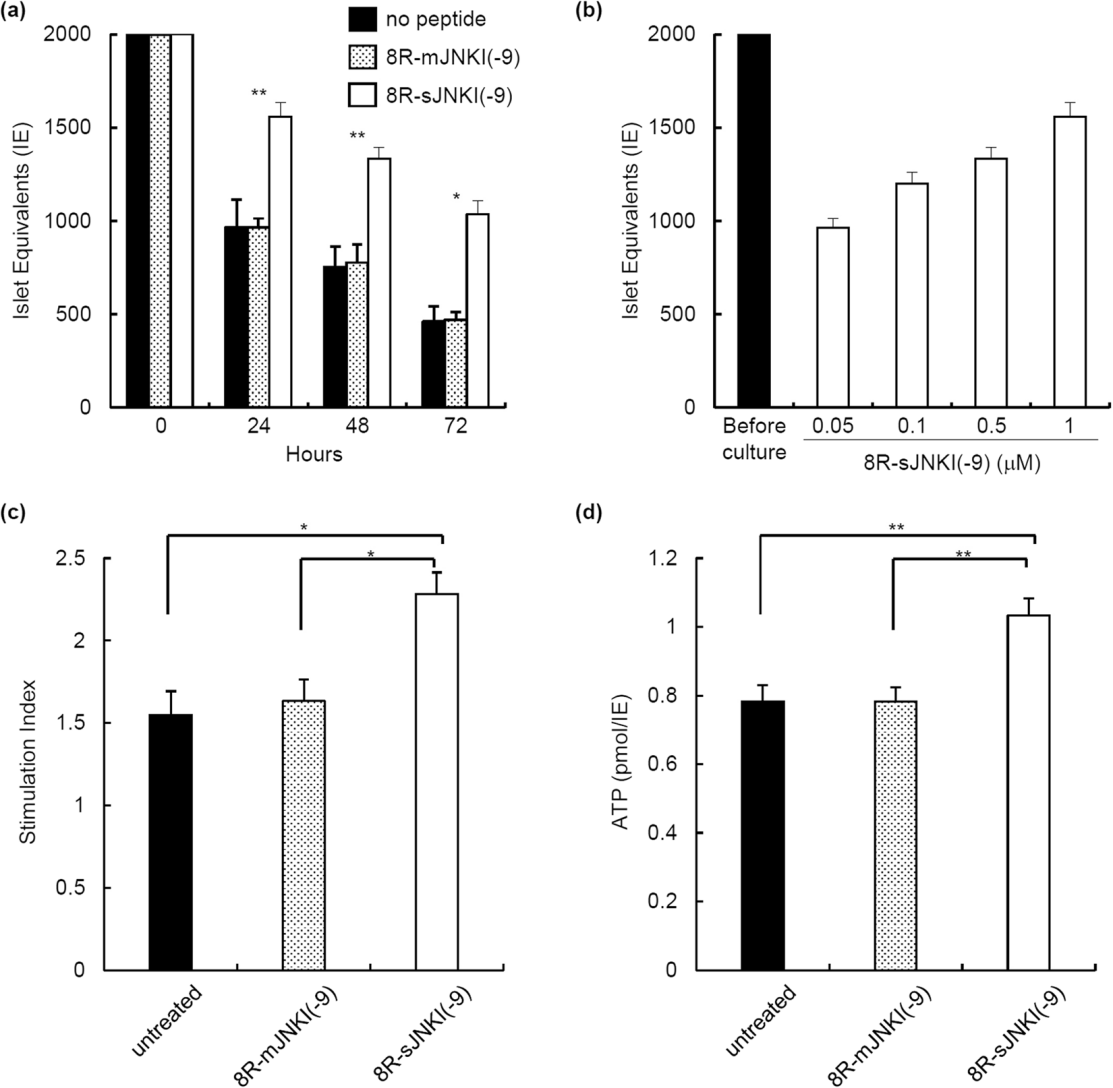
The function of isolated islets treated with 8R-sJNKI(-9). (**a**) The number of islets after culture. 2,000 IE of islets were treated with or without 8R-sJNKI(-9) or 8R-mJNKI(-9) for 72 h. After 24, 48, and 72 h of treatment, the cultured islets were counted to calculate the IEs in each group (n = 6). Asterisks indicate significant differences between the no peptide and 8R-sJNKI(-9) groups (*p < 0.01, **p < 0.05). **(b)** The dose-dependent prevention of the reduction in islet equivalents by 8R-sJNKI(-9). 2,000 IE of islets were incubated for 24 h with 0.05, 0.1, 0.5, or 1 µM of 8R-sJNKI(-9). After 24-h incubation, the islets were counted to calculate the IE in each group. **(c)** The stimulation index values of the isolated islets. The stimulation index was calculated by determining the ratio of insulin released from the islets in high-glucose media to the insulin released in low-glucose media. The data are expressed as the mean ± SE (each group; n = 6). **(d)** The cellular ATP content of the isolated islets. The ATP concentration of the cell lysate after preservation was measured using an ATP assay system. The data are expressed as the mean ± SE (each group; n = 6).

To evaluate the effect of 8R-sJNKI(-9) on islet culture, 2,000 IE of the isolated islets were cultured with or without 1 µM 8R-sJNKI(-9) or 8R-mJNKI(-9) for 24, 48, and 72 h. The number of islets treated with 8R-sJNKI(-9) was significantly higher than that of the untreated and 8R-mJNKI(-9) groups (Fig. 4a). 8R-sJNKI(-9) prevented the reduction in IE in a dose-dependent manner (Fig. 4b). These data suggest that treatment with 8R-sJNKI(-9) prevented a reduction in IE during culture.

To evaluate the islet function of each group *in vitro*, the stimulation index values of the islets were measured. The stimulation index values in the 8R-sJNKI(-9) group were significantly higher than those in the no peptide group and 8R-mJNKI(-9) group (no peptide group, 1.55 ± 0.14; 8R-sJNKI(-9) group, 1.63 ± 0.13; 8R-sJNKI(-9) group, 2.28 ± 0.13; n = 6 each; p < 0.01) (Fig. 4c). The ATP concentration of the cell lysate after culture was measured using an ATP assay system. The ATP content of 8R-sJNKI(-9) group (n = 6) was significantly higher than that of no peptide group (n = 6) and 8R-mJNKI(-9) group (n = 6) (no peptide group, 0.78 ± 0.05; 8R-sJNKI(-9) group, 0.78 ± 0.04; 8R-sJNKI(-9) group, 1.03 ± 0.05; n = 6 each; p < 0.01) (Fig. 4d).

### The *in vivo* assessment of isolated islets

**Figure 5:**
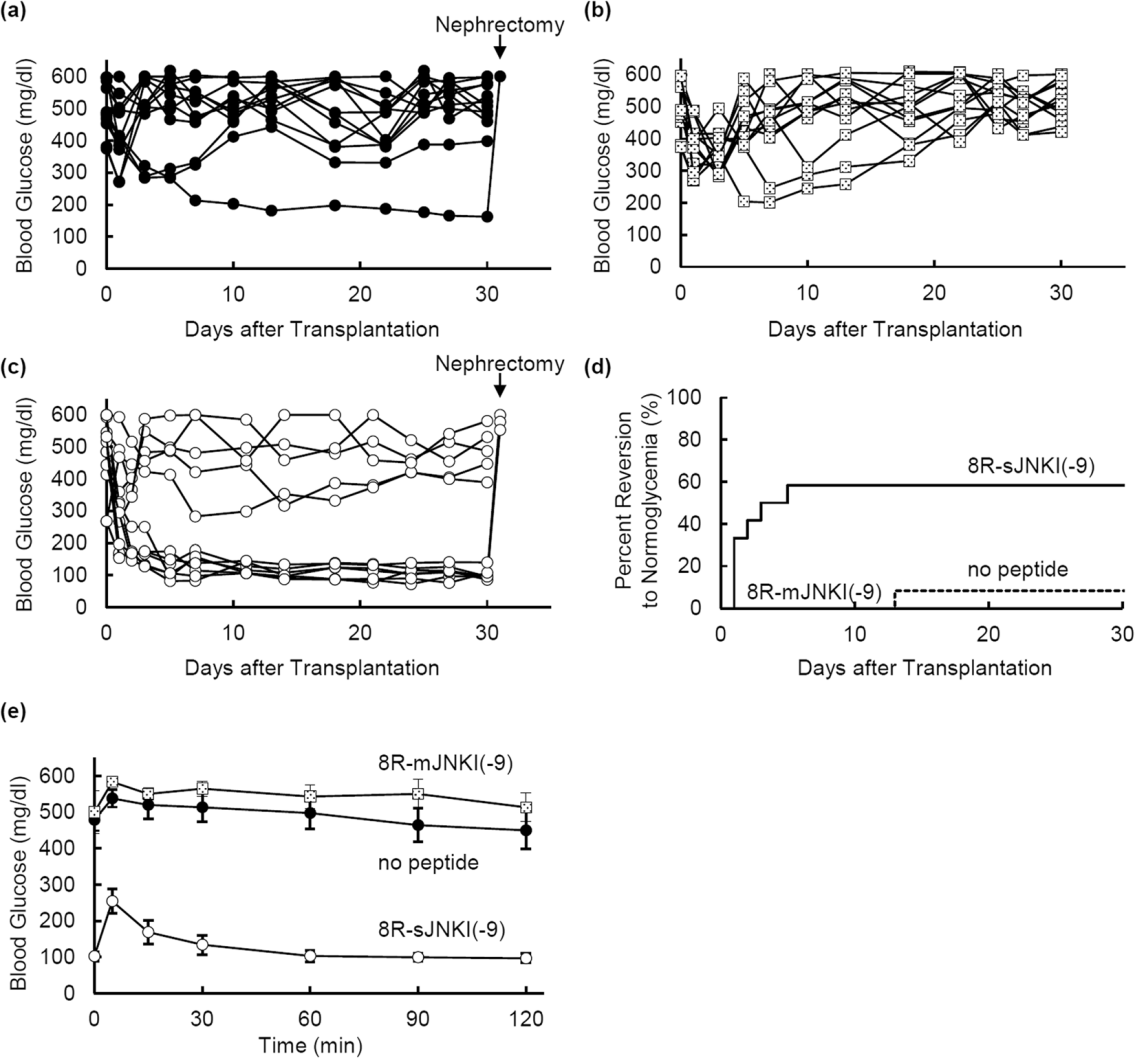
Transplantation of isolated islets into diabetic mice. Pig islets were treated with or without 1 µM 8R-sJNKI(-9) or 1 µM 8R-mJNKI(-9) for 6 h. A total of 1,500IE of the cultured islets were transplanted below the kidney capsule of immunodeficient diabetic mice. **(a)** The non-fasting blood glucose levels of mice that were transplanted with islets lacking JNK inhibitor treatment. **(b)** The non-fasting blood glucose levels of mice transplanted with islets that were treated with the 8R-mJNKI(-9). **(c)** The non-fasting blood glucose levels of mice transplanted with islets that were treated with the 8R-sJNKI(-9). **(d)** The percentage of diabetic mice with normoglycemia after islet transplantation. Normoglycemia was defined as two consecutive post-transplant blood glucose measurements of <200 mg/dl. Each group; n = 12 (n = 2 per one isolation) **(d)** The results of the IPGTT at 30 days after transplantation. The mice were fasted overnight before the test and intraperitoneally injected with glucose (2.0 g/kg body weight). The blood glucose levels were measured before and at 5, 15, 30, 60, and 120 minutes after injection. No peptide and 8R-mJNKI(-9) groups, n = 5 each (diabetic mice after islet transplantation); 8R-sJNKI(-9) group, n = 5 (normoglycemic mice after islet transplantation).

To evaluate whether or not treatment with 8R-sJNKI(-9) improves islet function, isolated islets with or without 1 µM 8R-sJNKI(-9) or 8R-mJNKI(-9) were incubated for 6 h. A total of 1,500 IE of the cultured islets were then transplanted below the kidney capsule of immunodeficient diabetic mice. The blood glucose levels of 7 of the 12 mice (58.3%) that received islets treated with 8R-sJNKI(-9) showed a gradual decrease and reached the normoglycemic range (Fig. 5c,d). The blood glucose levels returned to the pre-transplantation levels after the kidney-bearing islets were removed at 30 days post-transplantation (Fig. 5c). In contrast, only 1 of 12 mice (8.3%) that received islets treated with no peptide became normoglycemic (Fig. 5a,d). None of the 12 mice that received islets treated with 8R-mJNKI(-9) became normoglycemic (Fig. 5b,d). The difference in the attainment of post-transplantation normoglycemia was statistically significant (p < 0.05).

Intraperitoneal glucose tolerance testing (IPGTT) revealed that the fasting blood glucose levels of mice that received 8R-sJNKI-treated islets were lower than those of non-treated and 8R-mJNKI-treated mice before glucose injection and at 5, 15, 30, 60, 90, and 120 minutes after injection (p < 0.01) (Fig. 5e). These findings suggest that treatment with 8R-sJNKI(-9) improves islet function.

## Discussion

Organ preservation and the subsequent islet isolation led to the JNK activation strongly, and this has profound implications for apoptosis of pancreatic islets during and/or immediately after isolation^12,13,17,21,22^. In this study, we added 8R-sJNKI(-9) into the culture media that was transduced into the isolated islets. Our data showed that treatment with 8R-sJNKI(-9) before islet transplantation led to improved islet function at one tenth the concentration of 11R-JNKI. Previous studies showed that 17β-estradiol improved the survival of human islets after exposure of proinflammatory cytokines through the inhibition of JNK^23^ and that *Jnk1*-knockout islets secreted more insulin in response to glucose stimulation and were more resistant to cytokine-induced cell death than wild-type islets^24^. The JNK inhibition may be important for improving the outcomes of islet transplantation.

Our previous study showed that the addition of 10 µM 11R-JNKI into the culture media inhibited the activation of JNK and prevented islet apoptosis immediately after isolation^12^. On the other hand, this study showed that the treatment of 1 µM 8R-sJNKI(-9) induced a significant inhibition of JNK activation. Since the effect of 10 µM 8R-sJNKI(0) was similar to that of 10 µM 11R-JNKI, peptide transduction into cells may be similar between 11 R and 8 R + RPKR. The improvement of the efficacy of 8R-sJNKI(-9) may be due to the reduction in the peptide size and/or improvement of the inhibitory effect on sJNKI(-9).

In conclusion, the treatment with 8R-sJNKI(-9) improved islet yields and prevented the apoptosis of isolated islet cells. At 14 amino acids, the peptide length of 8R-sJNKI(-9) was smaller than that of 11R-JNKI and the concentration of 8R-sJNKI(-9) for JNK inhibition is sufficient at one tenth the concentration of 11R-JNKI, suggesting that 8R-sJNKI(-9) is more efficient and a lower priced JNK inhibitor than 11R-JNKI.

## Materials and Methods

### Peptide synthesis

Peptides (11R-JNKI, 8R-JNKI(0), 8R-sJNKI(-3), 8R-sJNKI(-6), 8R-sJNKI(-9), 8R-sJNKI(-12), 8R-mJNKI(-9); Fig. 1a) and FITC-conjugated peptides were synthesized by BIOSYNTHESIS (Lewisville, TX, USA). The peptides were purified by preparative reverse-phase HPLC and were >95% pure, with the expected amino acid composition and mass spectra.

### Transduction of the JNKI peptides

Porcine pancreatic islets (see “*Porcine pancreas procurement*, *preservation*, *and islet isolation*” section) were cultured with CMRL 1066 medium supplemented with 0.5% human serum albumin (HSA). The islets were incubated with 10 µM FITC-conjugated 11R-JNKI or 8R-sJNKI(-9) and examined using an Olympus confocal microscope.

### Inhibition of the JNK activity by the JNKI peptides

MIN6 cells (mouse β-cell-derived cell line) were incubated with 1–10 µM 11R-JNKI, 8R-sJNKIs, or 8R-mJNKI for 23 h and then incubated with 1 µg/ml anisomycin for 1 h. Activity of JNK was evaluated using a KinaseSTAR JNK Activity Assay Kit (BioVision Research Products, Mountain View, CA, USA), as previously described^12,13,17,25^.

### Specificity of 8R-sJNKI

JNK-inactivated MIN6 cells were generated by transfection of JNK siRNA (Thermo Fisher Scientific, Tokyo, Japan). Normal and JNK-inactivated MIN6 cells were cultured with or without 1 µM 8R-sJNKI(-9) for 23 h and then treated with pro-inflammatory cytokines (IL-1β 50 U/mL, TNF-α 1,000 U/mL, IFN-γ 1,000 U/mL; R&D Systems, Minneapolis, MN, USA) for 18 h. Cell viability was assessed as previously described^12,13,17^.

### Western blot analyses

Whole-cell extracts were fractionated by 10% SDS-PAGE and transferred to reinforced cellulose nitrate membrane. After blocking, the membranes were incubated overnight at 4 °C in TBS buffer (50 mmol/l Tris-HCl, 150 mmol/l NaCl) containing a 1:1,000 dilution of rabbit anti-phospho-c-Jun antibody (Cell Signaling, Danvers, MA, USA), mouse anti-JNK1 antibody (Cell Signaling), or mouse anti-β-actin antibody (Cell Signaling), and then incubated for 1 h at room temperature in TBS containing a 1:2,000 dilution of rabbit antibody or mouse antibody to IgG coupled to horseradish peroxidase (Cell Signaling). Immunoreactive bands were visualized by incubation with LumiGLO (Cell Signaling) and exposed to light-sensitive film.

### Porcine pancreas procurement, preservation, and islet isolation

Pancreata from 3-year-old pigs (female, n = 6) were used in this study. Pancreas procurement, preservation, and islet isolation was performed as previously described^3,12,13,17,26–29^. The warm ischemic time (WIT), cold ischemic time (CIT), Phase I period, and Phase II period were defined as previously described^3,12,13,17,26–29^. The islet score was defined as in Supplemental Table 1.

### Islet culture

Isolated islets was clutured with CMRL 1066 medium supplemented with 0.5% HSA for 6, 24, 48, and 72 h with or without 8R-mJNKI(-9) or 8R-sJNKI(-9).

### Islet evaluation

Dithizone staining, double fluorescein diacetate/propidium iodide (FDA/PI) staining, were performed as previously described^30–32^. Islet viability was also assessed after the separation of islet cells using a FACSAria. Isolated islets were cultured with or without 8R-sJNKI(-9) or 8R-mJNKI(-9) for 6 h. The separation of islet cells by Accutase (Innovative Cell Technologies, La Jolla, CA, USA) and PI/fluorescent annexin V staining were performed as previously described^12,13,17^.

To examine whether or not 8R-sJNKI(-9) can prevent islet apoptosis induced by exposure to cytokines, islets that had been cultured for 48 h were treated with or without 8R-sJNKI(-9) or 8R-mJNKI(-9) for 23 h and then exposed to a cocktail of cytokines (IL-1β 50 U/mL, TNF-α 1,000 U/mL, IFN-γ 1,000 U/mL) for 18 h. Cell viability was assessed as previously described^12,13,17^.

### The determination of ATP production

To evaluate the production of adenosine triphosphate (ATP), pancreatic islets were incubated overnight with culture medium and solubilized. An ATP assay system (Toyo Inki, Tokyo, Japan) was used to measure the ATP content. After allowing the reagents to equilibrate to room temperature, 10 µL of cell extracts were added to 100 µL of the reagents. The samples were measured using a luminometer.

### The insulin secretory response

The insulin secretory response of isolated islets during glucose stimulation was evaluated according to a procedure described by Shapiro *et al*.^1^. The data were normalized to total DNA^17^.

### The *in vivo* assessment

Isolated islets were incubated with or without 1 µM 8R-sJNKI(-9) or 1 µM 8R-mJNKI(-9) for 6 h. A total of 1,500 IE of the cultured islets were processed for transplantation. Diabetes induction, transplantation into SCID mice (CLEA Japan, Inc. Meguro, Tokyo), and IPGTT were performed as previously described^12,13,17,18^. All of the animal studies were approved by the Institutional Animal Care and Use Committee of the University of the Ryukyus.

### Statistical analyses

All data were expressed as the mean ± SE. Student’s *t*-test was used two compare two samples from independent groups, and was performed using the Microsoft Excel software program. To compare the data among the groups, a repeated measures ANOVA was used. The differences in the duration of graft survival between the groups were evaluated using the Kaplan–Meier log-rank test, which was performed using the StatView software program. A p-value of <0.05 was considered to indicate statistical significance.

All methods were performed in accordance with the relevant guidelines and regulations.

## Electronic supplementary material

Supplemental Data 1
