# Supplementary material for: Modified cell-permeable JNK inhibitors efficiently prevents islet apoptosis and improves the outcome of islet transplantation
Source: Sci Rep. 2018 Jul 23;8:11082. doi: 10.1038/s41598-018-29481-9 (PMC6056537; doi:10.1038/s41598-018-29481-9)
Supplement: Supplementary file 1 — Supplemental Data 1 [file 41598_2018_29481_MOESM1_ESM.pdf]

Original Article

Modified cell-permeable JNK inhibitors efficiently prevents islet apoptosis and improves the outcome of islet transplantation

Hirofumi Noguchi [1,*], Chika Miyagi-Shiohira [1], Yoshiki Nakashima [1], Nana Ebi [1], Eri Hamada [1], Yoshihito Tamaki [1], Kazuho Kuwae [1], Naoya Kobayashi [2], Issei Saitoh [3], Masami Watanabe [4]

1. Department of Regenerative Medicine, Graduate School of Medicine, University of the Ryukyus, Okinawa 903-0215, Japan

2. Okayama Saidaiji Hospital, Okayama 704-8192, Japan

3. Division of Pediatric Dentistry, Graduate School of Medical and Dental Science, Niigata University, Niigata 951-8514, Japan

4. Department of Urology, Okayama University Graduate School of Medicine, Dentistry and Pharmaceutical Sciences, Okayama 700-8558, Japan

***Address correspondence to:**

Hirofumi Noguchi, MD, PhD

Department of Regenerative Medicine

Graduate School of Medicine, University of the Ryukyus

207 Uehara, Nishihara, Okinawa 903-0215, Japan.

Tel: +81-98-895-3331; Fax: +81-98-895-3331

E-mail: noguchih@med.u-ryukyu.ac.jp

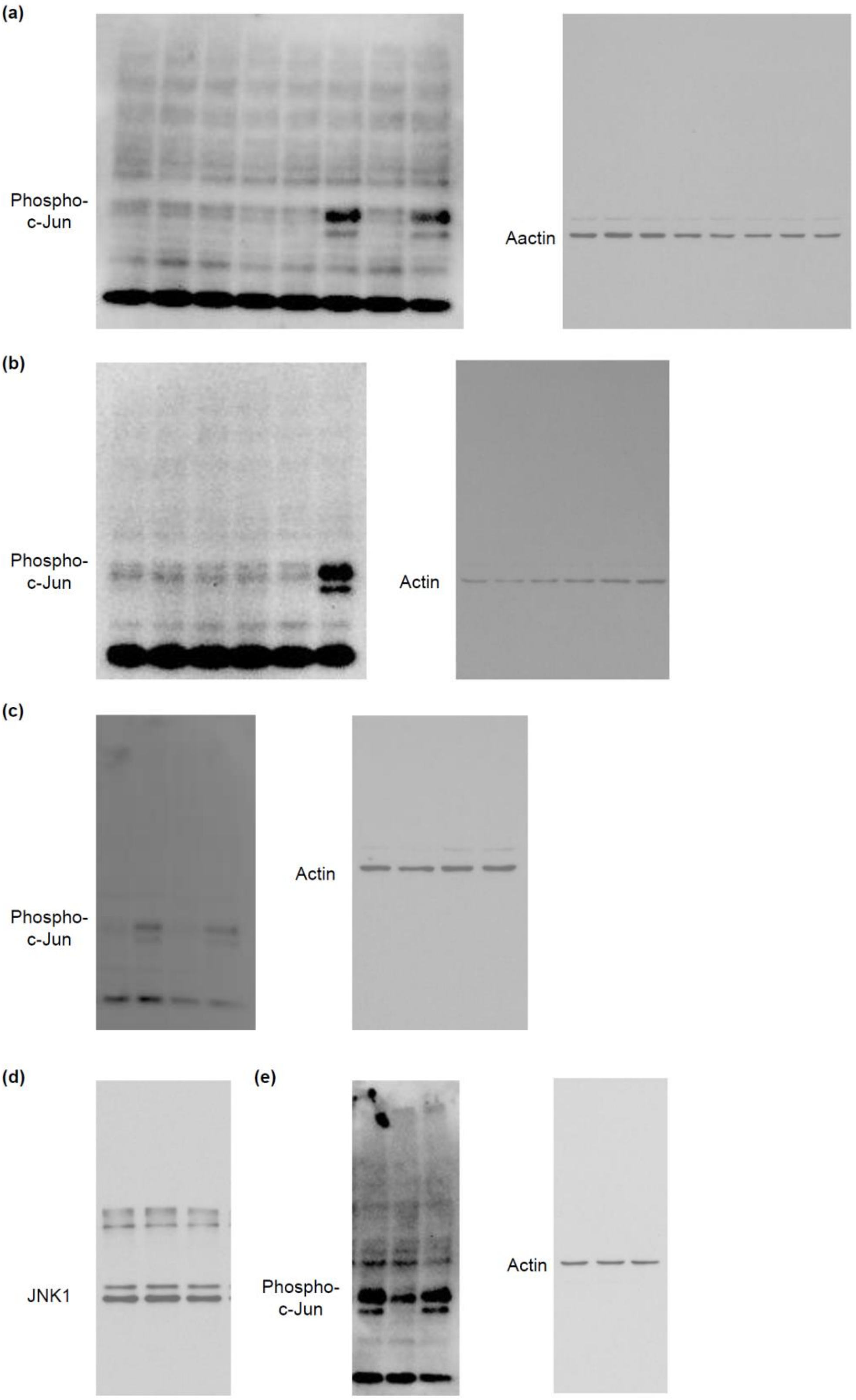
(a)
Phospho-
c-Jun
Aactin
(b)
Phospho-
c-Jun
Actin
(c)
Actin
Phospho-
c-Jun
(d)
JNK1
(e)
Phospho-
c-Jun
Actin

Supplemental Figure 1. Full-length gels

(a) Full-length gels of Figure 1c (Phospho-c-Jun and Actin). (b) Full-length gels of Figure 1d (Phospho-c-Jun and Actin). (c) Full-length gels of Figure 2a (Phospho-c-Jun and Actin). (d) Full-length gels of Figure 3a (JNK1). (e) Full-length gels of Figure 3a (Phospho-c-Jun and Actin).

Supplemental Table 1. Islet score

	0	1	2
Shape	Flat	Between	Spherical
Border	Irregular	Between	Well-rounded
Integrity	Fragmented	Between	Solid/compact
Uniformity of Stain	Not uniform	Between	Perfectly Uniform
Size	All<100μm	Between	10%>200μm
